# Stent thrombosis and adverse cardiovascular outcomes observed between six months and five years with sirolimus-eluting stents and other drug-eluting stents in patients with Type 2 diabetes mellitus complicated by coronary artery disease: A systematic review and meta-analysis

**DOI:** 10.1097/MD.0000000000004130

**Published:** 2016-07-08

**Authors:** Pravesh Kumar Bundhun, M. Zafooruddin Sani Soogund, Manish Pursun, Meng-Hua Chen

**Affiliations:** a Institute of Cardiovascular Diseases, the First Affiliated Hospital; b Guangxi Medical University, Nanning, Guangxi, PR China.

**Keywords:** adverse cardiovascular outcomes, coronary artery diseases, drug-eluting stents, sirolimus-eluting stents, stent thrombosis, type 2 diabetes mellitus

## Abstract

This study aimed to compare 6 months to 5 years stent thrombosis (ST) and adverse cardiovascular outcomes associated with sirolimus-eluting stents (SES) and other drug-eluting stents (DES) in patients with type 2 diabetes mellitus (T2DM).

Electronic databases were searched for studies comparing SES with other DES in patients with T2DM. Total ST, definite ST, probable ST, and other adverse cardiovascular outcomes reported between 6 months and 5 years were considered as the clinical end points in this study. Odds ratios (ORs) with 95% confidence intervals (CIs) were calculated for categorical variables and the pooled analyses were performed with RevMan 5.3 software.

Twenty-nine studies involving a total number of 25,729 patients with diabetes were included in this meta-analysis. SES were not associated with significantly higher total, definite, and probable STs with OR: 0.95, 95% CI: 0.77–1.17, *P* = 0.62; OR: 0.94, 95% CI: 0.65–1.37, *P* = 0.76; and OR: 1.05, 95% CI: 0.77–1.45, *P* = 0.74, respectively. SES were also noninferior to the other non-sirolimus eluting drug eluting stents (non-SE DES) in terms of all-cause mortality, cardiac death, myocardial infarction, and stroke with OR: 0.92, 95% CI: 0.82–1.03, *P* = 0.16; OR: 1.09, 95% CI: 0.88–1.35, *P* = 0.44; OR: 0.92, 95% CI: 0.80–1.06, *P* = 0.26; and OR: 0.79, 95% CI: 0.49–1.28, *P* = 0.43, respectively. Target vessel revascularization, target lesion revascularization, and major adverse cardiac events were also similarly reported between SES and non-SE DES with OR: 1.04, 95% CI: 0.83–1.31, *P* = 0.72; OR: 1.25, 95% CI: 0.95–1.64, *P* = 0.11; and OR: 1.06, 95% CI: 0.90–1.25, *P* = 0.49, respectively.

During this particular follow-up period, SES were not associated with any increase in ST among these patients with T2DM. Mortality and other adverse cardiovascular outcomes were also not significantly different between these 2 groups. Hence, SES should be considered neither superior nor inferior to other DES. They are expected to be equally effective and safe to use in patients with T2DM.

## 1 Introduction

Percutaneous coronary intervention with drug-eluting stents (DES) is becoming more demanding year by year, especially among patients with diabetes with coronary artery diseases.^[1]^ Even if the revascularization rate has significantly decreased in patients with diabetes treated by DES,^[2]^ stent thrombosis (ST) is still a major concern in these patients.^[3]^ Recently, controversies were observed when different types of individual DES were compared. In patients with type 2 diabetes mellitus (T2DM), several studies showed sirolimus-eluting stents (SES) to be more effective compared to paclitaxel-eluting stents (PES).^[4]^ However, other studies showed SES and PES to be comparable.^[5]^ When SES were compared to everolimus-eluting stents (EES), EES were associated with better outcomes in patients with T2DM.^[6]^ However, in other studies EES were noninferior to SES.^[7]^ It is believed that different follow-up periods reported in several cohorts could indirectly have contributed to these controversies. Therefore, this study aimed to compare 6 months to 5 years ST and other adverse cardiovascular outcomes associated with SES and other DES, referred in this study as “non-SE DES,” using a larger number of patients with diabetes.

## 2 Methods

### 2.1 Data sources and search strategies

PubMed, Medline, EMBASE, and the Cochrane library were searched for randomized controlled trials and observational studies comparing SES with other DES in patients with diabetes by tying the words or phrase “sirolimus eluting stents and drug eluting stents and diabetes mellitus.” The word “drug eluting stents” was later replaced by the specific names of other DES such as “paclitaxel eluting stents, everolimus eluting stents and zotarolimus eluting stents.” To further enhance this search, abbreviations of the above-mentioned words such as “SES, DES, PES, EES, ZES” were also used. Reference lists of most suitable articles were also checked for relevant studies. This search was restricted only to articles published in English.

### 2.2 Inclusion and exclusion criteria

Studies were included if:

- They were randomized controlled trials or observational studies.
- They compared SES with non-SE DES in patients with T2DM.
- They reported ST and/or other adverse cardiovascular outcomes observed between SES and non-SE DES.
- They had a follow-up period between 6 months and 5 years.

Studies were excluded if:

- They were meta-analyses, case studies, or letters to editors.
- They did not compare SES with non-SE DES in patients with T2DM.
- They did not report ST and/or other adverse cardiovascular outcomes observed between SES and other DES.
- They had a follow-up period of <6 months.
- They were associated with the same trial or they were duplicates.

### 2.3 Outcomes and follow-ups

This study assessed 6 months to 5 years ST and other cardiovascular outcomes in patients with diabetes treated by SES and non-SE DES. The end points analyzed in this study included:

- ST that was defined according to the Academic Research Consortium^[8]^ and involved:
  - Total ST
  - Definite ST
  - Probable ST
- All-cause mortality
- Cardiac mortality
- Myocardial infarction (MI)
- Target vessel revascularization (TVR)
- Target lesion revascularization (TLR)
- Stroke
  Major adverse cardiac events (MACEs) that consisted of death, MI, and revascularization (composite end point, which consisted of death, MI, and ST, was reported in only 1 study and was therefore considered in the same category as MACEs)

Patients were followed for a period ranging from 6 months to 5 years. However, ST was also analyzed during a follow-up period ranging from 6 months to 2 years and a follow-up period of >2 years. The outcomes reported in each study along with their follow-up periods have been summarized in Table 1.

**Table 1:**
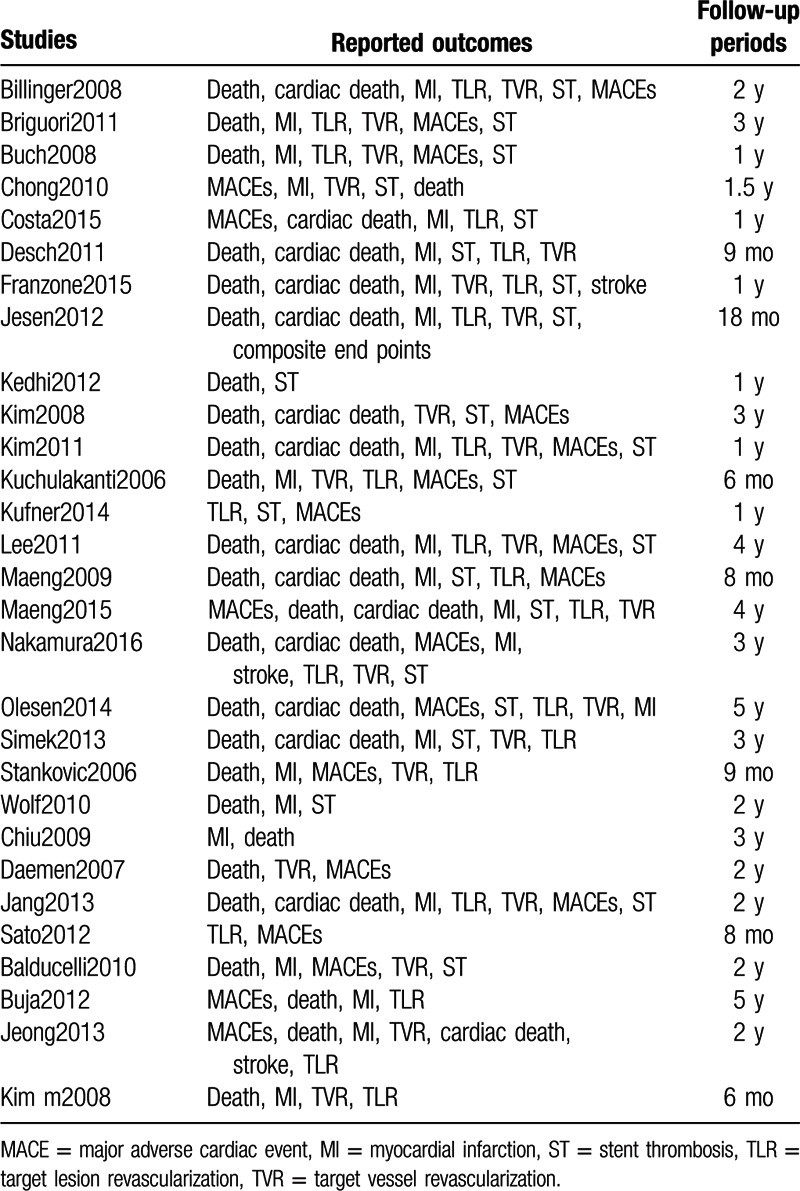
Reported outcomes and follow-up periods.

According to Table 1, ST was reported in >20 studies, whereas all-cause mortality was reported in 26 studies. When ST was further subdivided, definite ST was reported in 12 studies, whereas only 9 studies reported probable ST. MI was reported in 25 studies and MACEs were reported in 24 studies. TVR and TLR were reported in 22 studies each and stroke was reported in only 3 studies.

### 2.4 Data extraction and review

Three authors (PKB, MZSS, and MP) independently reviewed the studies that were selected for this meta-analysis and then assessed whether these articles were fully eligible. The type of study reported, data concerning the total number of patients with T2DM treated by SES and the other DES, respectively, the different types of DES involved, data concerning the baseline features of the patients included, the reported outcomes, as well as the corresponding follow-up periods were carefully extracted. This was not an easy task for the authors; therefore, any disagreement or confusion about including certain studies or data was carefully discussed in order to finally reach a decision. However, if a final decision could not be reached, the fourth author (MHC) was contacted to solve this issue. The bias risk was assessed using the 6 main components recommended by the Cochrane Collaboration.^[9]^

### 2.5 Statistical analysis

The Preferred Reporting Items for Systematic Reviews and Meta-Analyses were considered in this study.^[10]^ Assessment of heterogeneity during the subgroup analysis was performed using the following:

- Cochrane Q-statistic test:
  whereby a “*P* value” ≤0.05 was considered statistically significant and a “*P* value” >0.05 was considered statistically insignificant
- Cochrane *I*^2^-statistic test:

whereby an *I*^2^ value of 0% indicated no heterogeneity, and an increased heterogeneity was represented by a larger value (an *I*^2^ value of <25% indicated a low heterogeneity, an *I*^2^ value ranging from 25% to 50% represented a moderate heterogeneity, and an *I*^2^ value of >50% indicated a higher heterogeneity)

If *I*^2^ was ≤50%, a fixed effect model was used during the statistical analysis. However, if *I*^2^ was >50%, a random effect model was used.

Funnel plots were assessed for publication bias. Odds ratios (ORs) with 95% confidence intervals (CIs) were calculated for categorical variables. The pooled analyses were performed with RevMan 5.3 software.

Ethical approval was not necessary for this type of study that involved data obtained from randomized trials and observational cohorts.

## 3 Results

### 3.1 Search results

**Figure 1:**
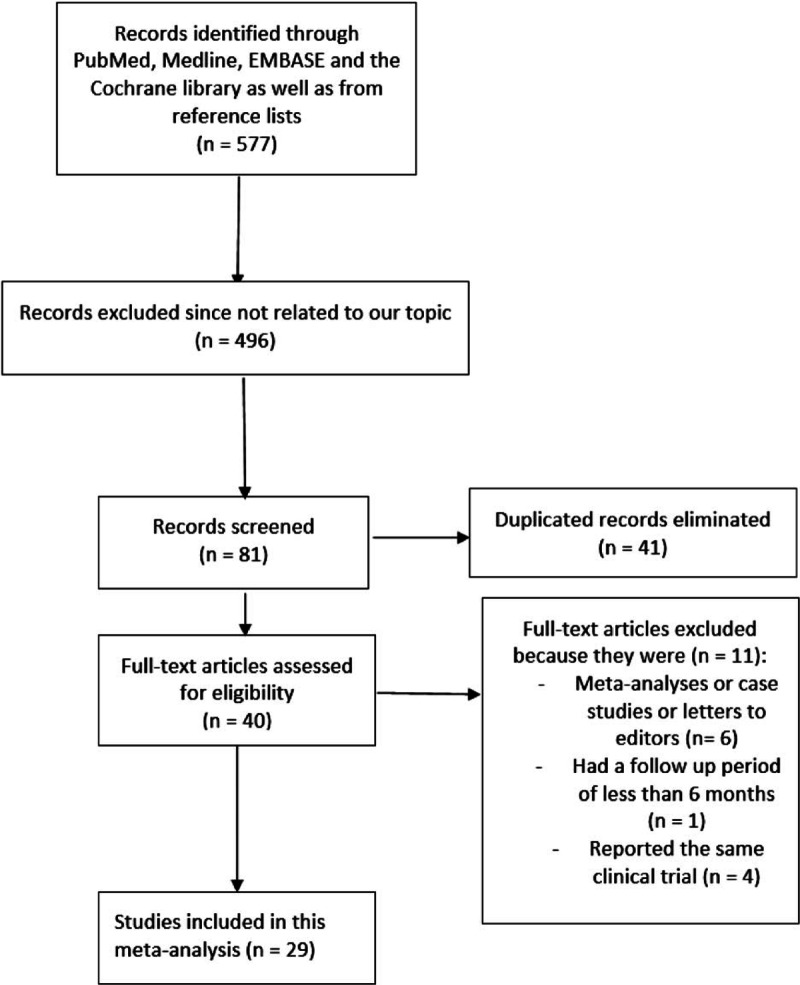
Flow diagram representing the study selection.

A total number of 577 articles were obtained from PubMed, Medline, EMBASE, the Cochrane library, and the reference lists of suitable articles. A total of 496 articles were eliminated after a careful assessment of the titles and abstracts because they were not related to the topic of this research. Another 41 articles were eliminated because they were duplicates. Forty full-text articles were assessed for eligibility. Eleven articles were further eliminated because 2 articles were meta-analyses, 4 articles were case studies or letters addressed to editors, 1 study reported a follow-up period of <6 months, and 4 studies were associated with the same trials. Finally, 29 articles were selected and included in this meta-analysis. Fig. 1 represents the flow diagram for the study selection.

### 3.2 General features of the studies included

Twenty-nine studies (12 trials and 17 observational studies) involving a total number of 25,729 patients with T2DM (10,520 patients were treated by SES and 15,209 patients were treated by non-SS DES) were included in this meta-analysis. SES were compared with PES, EES, and zotarolimus-eluting stents (ZES). Twenty-three studies compared SES with PES. Nine studies compared SES with EES, whereas only 4 studies compared SES with ZES in these patients with T2DM. Study Kedhi2012 involved the largest number of patients treated by SES and non-SS DES. The general features of the studies included in this meta-analysis have been summarized in Table 2.

**Table 2:**
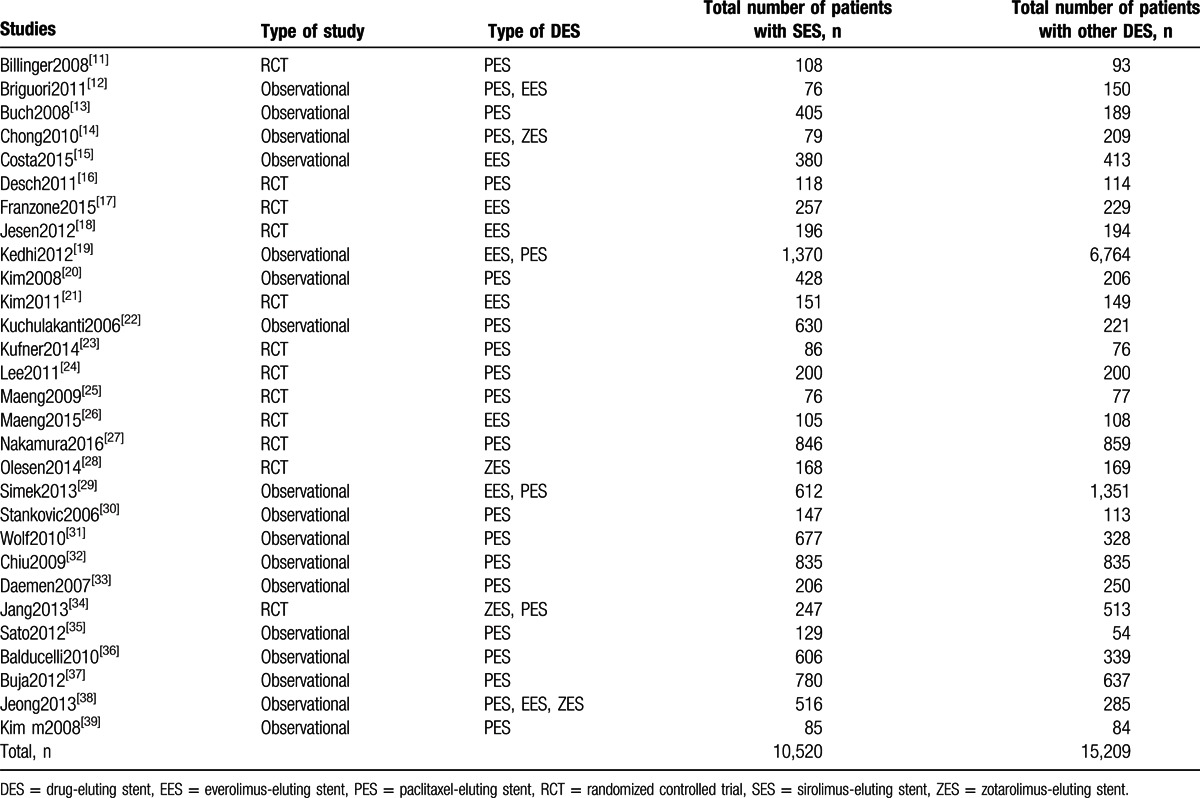
General features of the studies included.

### 3.3 Baseline features of the studies included

The baseline characteristics of the studies included in this meta-analysis have been summarized in Table 3.

**Table 3:**
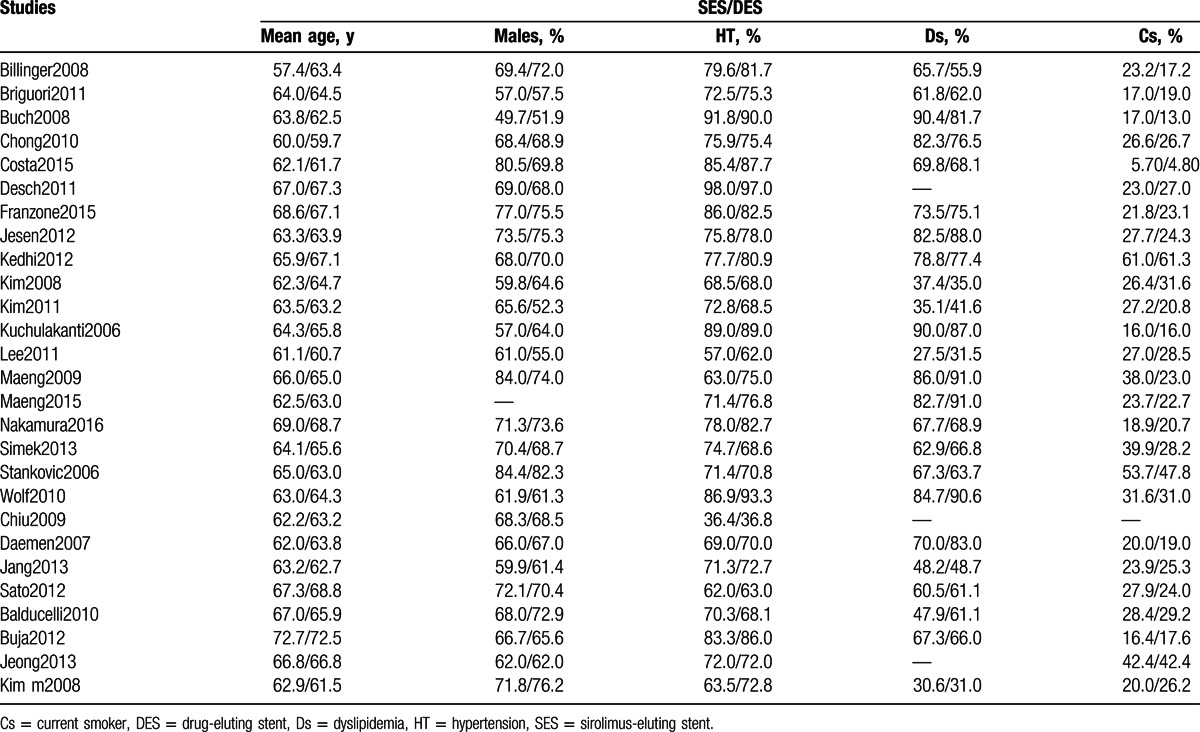
Baseline features of the studies included in this meta-analysis.

Studies not included in Table 3 did not report any baseline feature in their original manuscript and were therefore ignored.

A mean age ranging from 57.4 to 72.7 years was reported among the patients. More details concerning the percentage of males in each study and groups, patients with hypertension, dyslipidemia, and the percentage of patients who smoke have all been listed in Table 3. According to Table 3, there was no significant difference in the baseline features among patients treated by SES and patients treated by non-SE DES.

Table 4 lists the percentage of patients on insulin therapy.

**Table 4:**
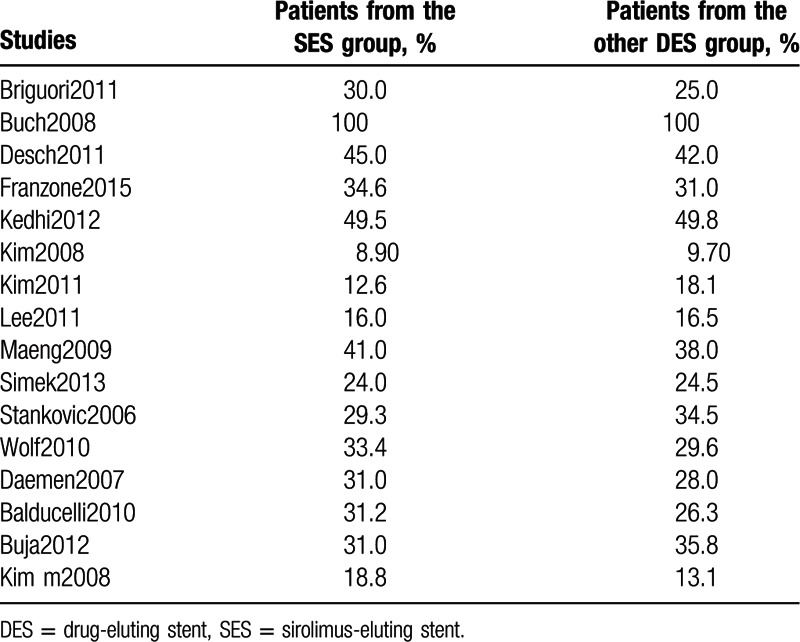
Patients on insulin therapy.

Studies not included in Table 4 did not report the number of patients on insulin therapy and they have therefore been ignored.

According to Table 4, study Buch2008 involved 100% of patients with insulin-treated T2DM in the SES and non-SE DES groups, whereas study Kedhi2012 reported 45% of patients in the SES group who were on insulin therapy and 42% patients in the non-SE DES group treated by insulin therapy. Details involving insulin treatment have been given in Table 4.

### 3.4 Stent thrombosis associated with SES and non-SE DES

Table 5 summarizes the results of this meta-analysis.

**Table 5:**
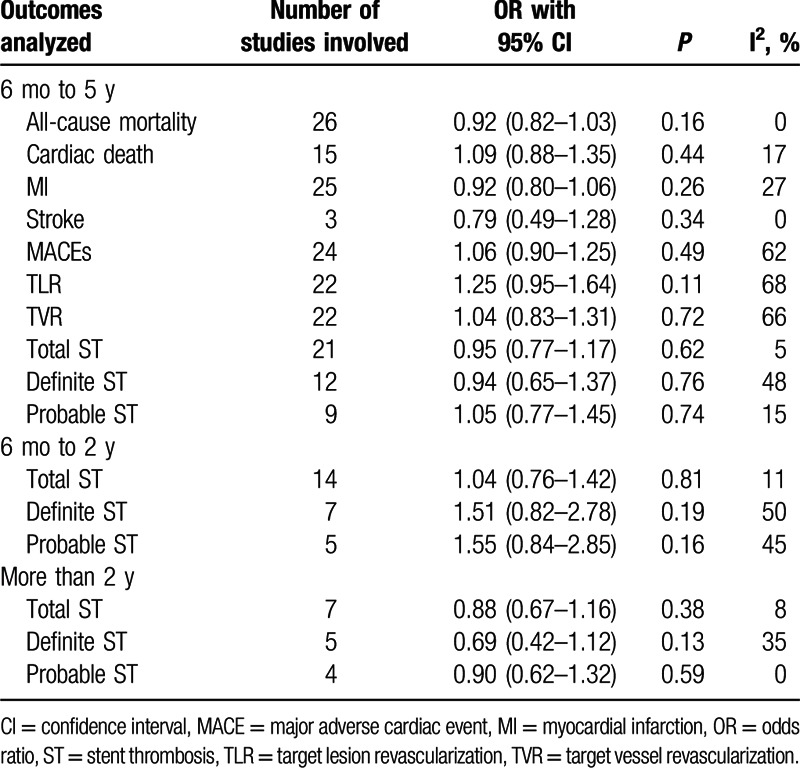
Results of this meta-analysis.

**Figure 2:**
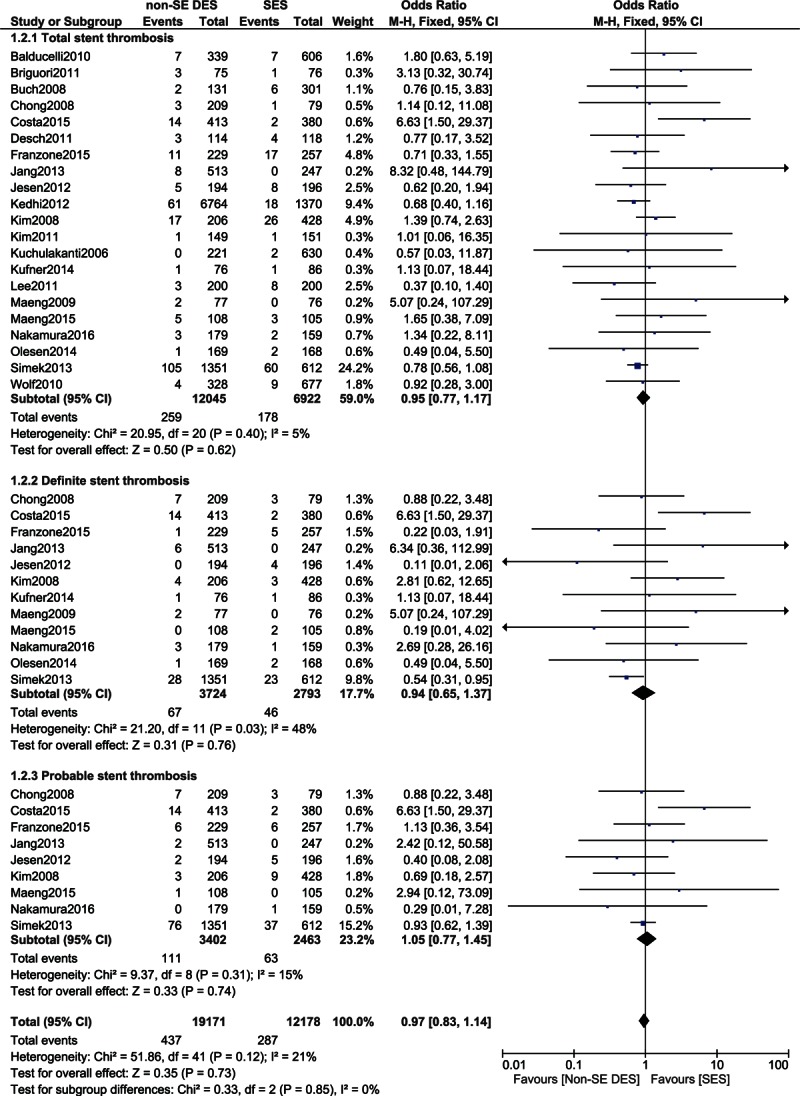
Comparing stent thrombosis between SES and non-SE DES during a follow-up period ranging from 6 months to 5 years. CI = confidence interval, DES = drug-eluting stent, df = degree of freedom, SES = sirolimus-eluting stent.

Between 6 months and 5 years, SES were not associated with significantly higher total, definite, and probable STs with OR: 0.95, 95% CI: 0.77–1.17, *P* = 0.62; OR: 0.94, 95% CI: 0.65–1.37, *P* = 0.76; and OR: 1.05, 95% CI: 0.77–1.45, *P* = 0.74, respectively, compared to non-SE DES. SES were noninferior to non-SE DES in these patients with T2DM. Results comparing 6 months to 5 years ST have been represented in Fig. 2.

Six months to 2 years ST and ST >2-year follow-up were also analyzed.

**Figure 3:**
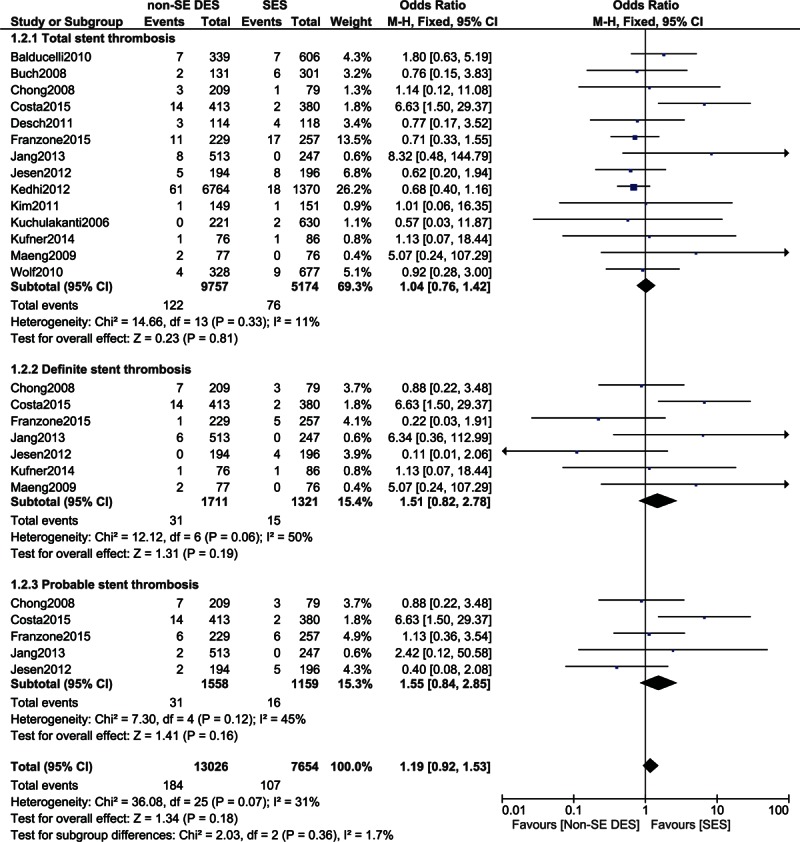
Comparing stent thrombosis between SES and non-SE DES during a follow-up period ranging from 6 months to 2 years. CI = confidence interval, DES = drug-eluting stent, df = degree of freedom, SES = sirolimus-eluting stent.

From 6 months to 2 years, total ST was noninferior between these 2 groups with OR: 1.04, 95% CI: 0.76–1.42, *P* = 0.81. Definite and probable STs were also similarly reported with OR: 1.51, 95% CI: 0.82–2.78, *P* = 0.19 and OR: 1.55, 95% CI: 0.84–2.85, *P* = 0.16, respectively, in these patients with T2DM. These results have been represented in Fig. 3.

**Figure 4:**
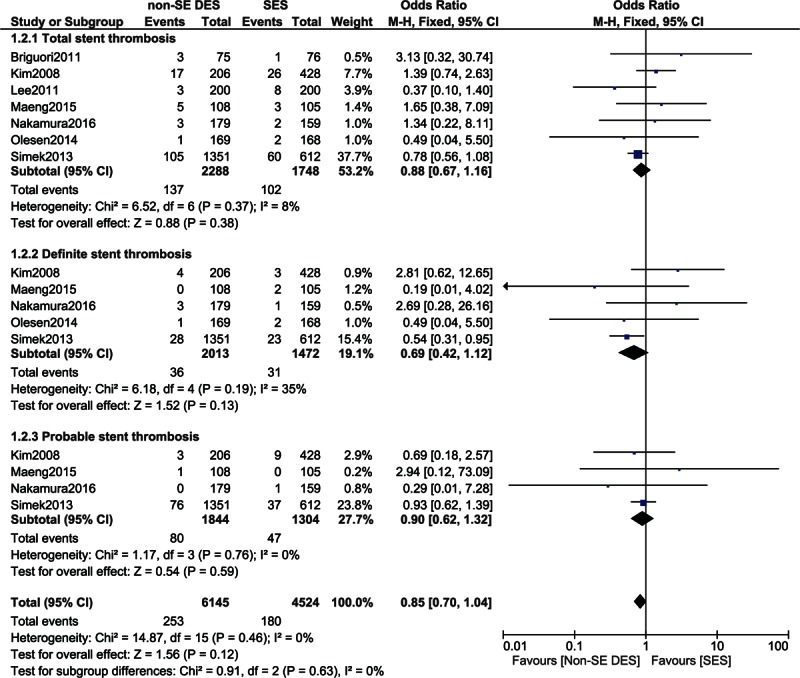
Comparing stent thrombosis between SES and non-SE DES during a follow-up period of >2 years. CI = confidence interval, DES = drug-eluting stent, df = degree of freedom, SES = sirolimus-eluting stent.

After 2 years, total, definite, and probable STs were not significantly different in these patients with diabetes with OR: 0.88, 95% CI: 0.67–1.16, *P* = 0.31; OR: 0.69, 95% CI: 0.42–1.12, *P* = 0.13; and OR: 0.90, 95% CI: 0.62–1.32, *P* = 0.59, respectively. These results have been illustrated in Fig. 4.

### 3.5 Adverse cardiovascular outcomes associated with SES and non-SE DES

**Figure 5:**
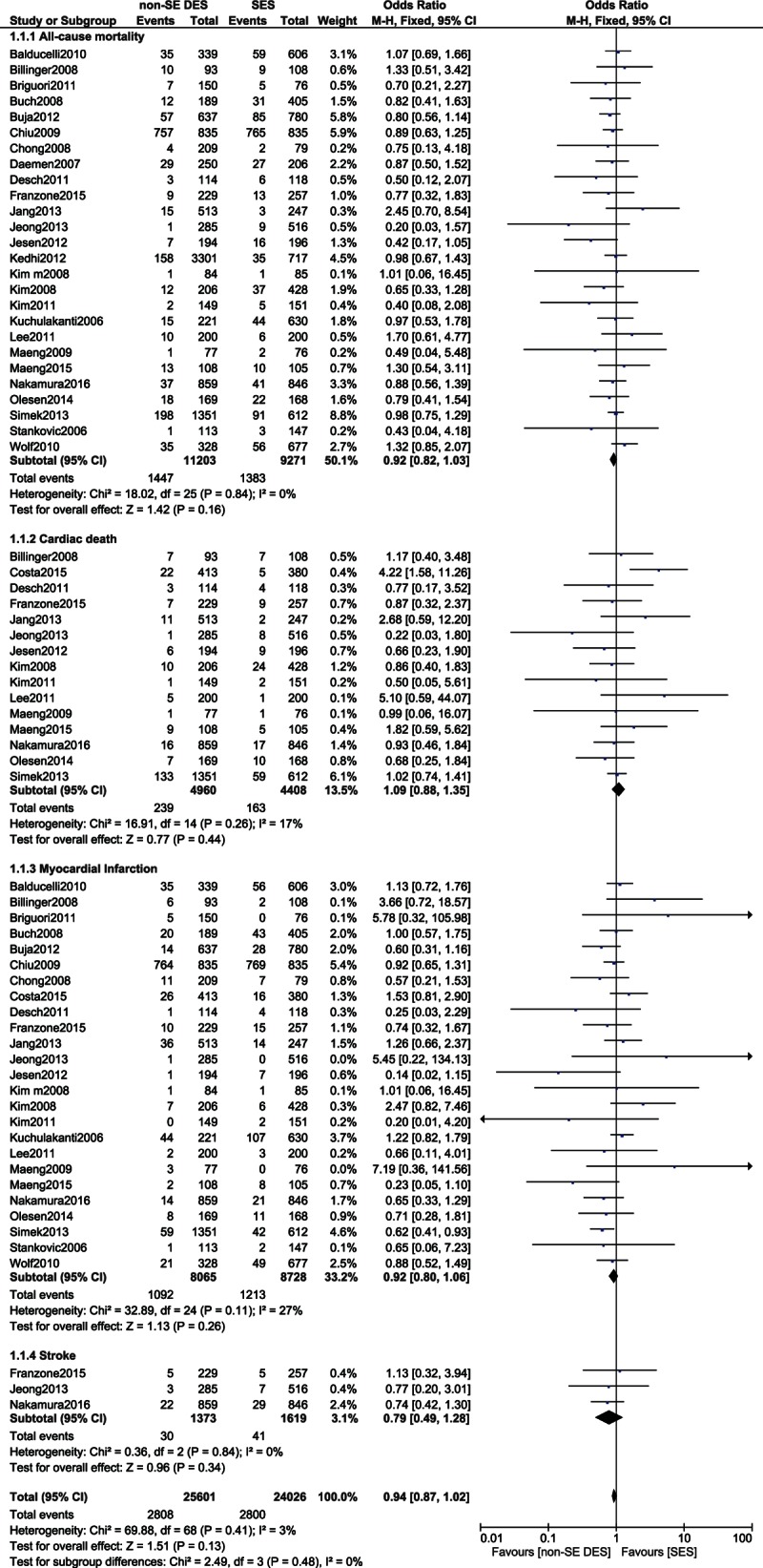
Comparing the adverse cardiovascular outcomes (part 1). CI = confidence interval, DES = drug-eluting stent, df = degree of freedom, SES = sirolimus-eluting stent.

Six months to 5 years adverse cardiovascular outcomes associated with SES and non-SE DES were also compared in these patients with T2DM. SES were noninferior to the other non-SE DES in terms of all-cause mortality, cardiac death, MI, and stroke with OR: 0.92, 95% CI: 0.82–1.03, *P* = 0.16; OR: 1.09, 95% CI: 0.88–1.35, *P* = 0.44; OR: 0.92, 95% CI: 0.80–1.06, *P* = 0.26; and OR: 0.79, 95% CI: 0.49–1.28, *P* = 0.43, respectively. These results have been shown in Fig. 5.

**Figure 6:**
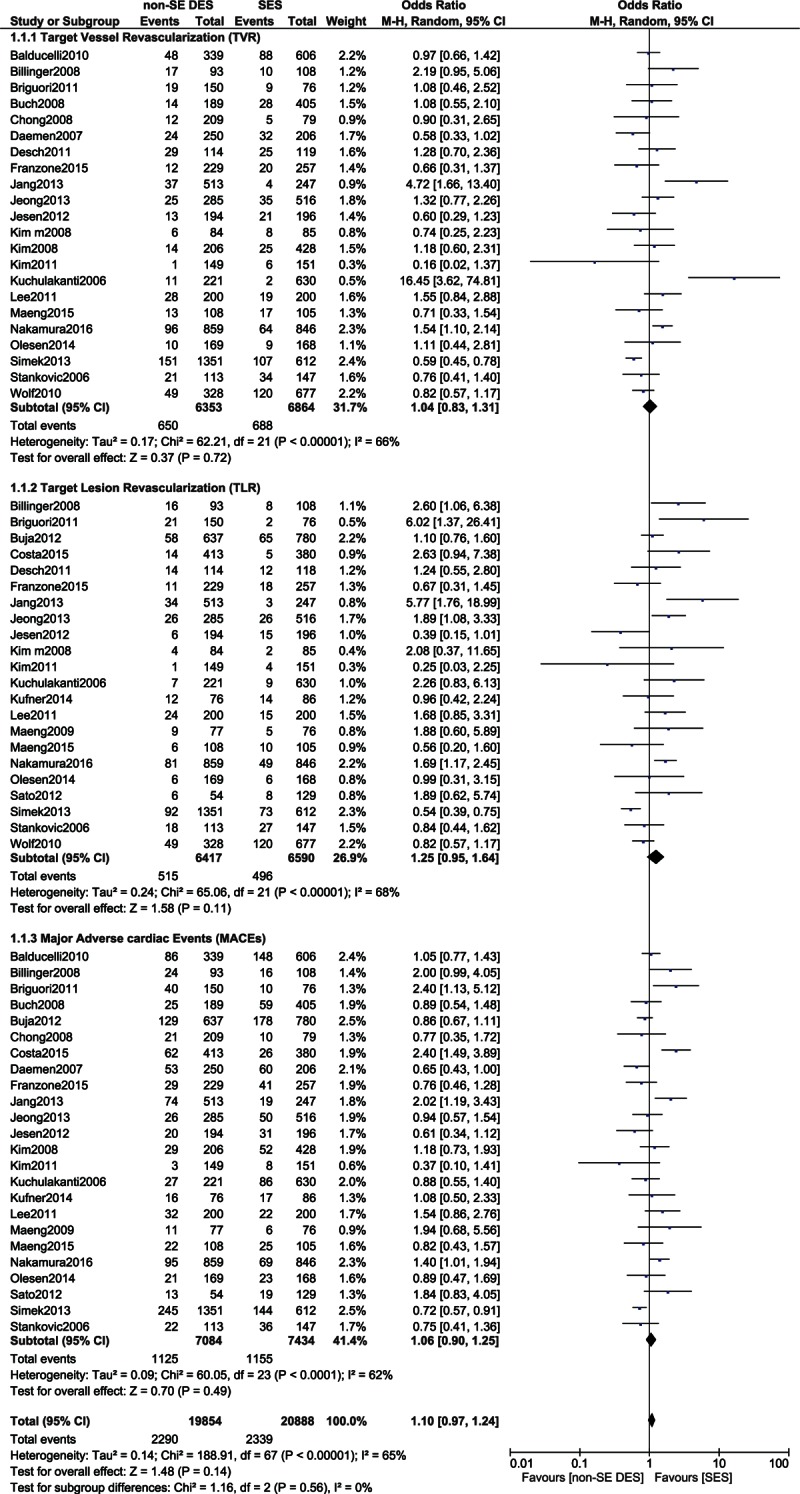
Comparing the adverse cardiovascular outcomes (part 2). CI = confidence interval, DES = drug-eluting stent, df = degree of freedom, SES = sirolimus-eluting stent.

TVR, TLR, and MACEs were also similarly reported with SES and non-SS DES, with OR: 1.04, 95% CI: 0.83–1.31, *P* = 0.72; OR: 1.25, 95% CI: 0.95–1.64, *P* = 0.11; and OR: 1.06, 95% CI: 0.90–1.25, *P* = 0.49, respectively. These results have been illustrated in Fig. 6.

### 3.6 Sensitivity analysis

**Figure 7:**
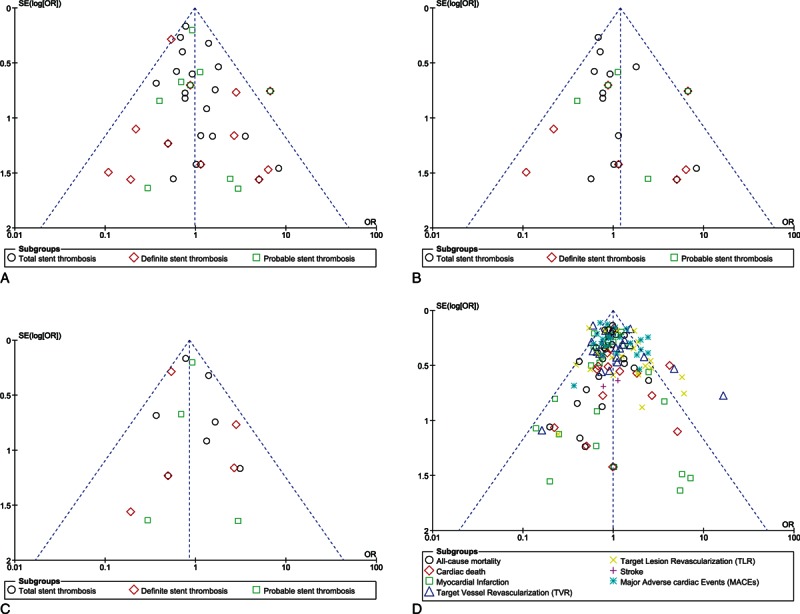
(A–D) Funnel plots assessing sensitivity analysis. OR = odds ratio.

After visually assessing the funnel plots (Fig. 7A–D), a low or moderate publication bias was observed among several subgroups analyzing ST in these patients with T2DM. However, when analyzing the other cardiovascular outcomes, an increased risk of bias was observed in certain but not all of the subgroups.

## 4 Discussion

Controversies have been observed when comparing SES with other DES such as PES, EES, or ZES in patients with T2DM. This analysis showed that SES were neither inferior nor superior to non-SE DES in patients with T2DM. Between 6 months and 5 years, total, definite, and probable STs were not significantly different in these 2 groups. Other adverse cardiovascular outcomes such as mortality, cardiac death, MI, stroke, TVR, TLR, and MACEs were also similarly manifested among patients treated with SES and non-SE DES.

Similarly, the meta-analysis involving 11,000 patients with T2DM showed no significant difference in MACEs reported between SES and PES.^[40]^ Moreover, another meta-analysis involving 7370 patients obtained from 5 randomized trials showed no significant difference between EES and SES in terms of safety and clinical efficacy.^[7]^ The results were consistent with the current analysis, even though PES, EES, and ZES were combined together.

In contrast, Bundhun et al recently showed EES to be associated with significantly better adverse clinical outcomes in patients with diabetes when compared with non-EE DES.^[6]^ However, a 1-year follow-up period might not be sufficient to analyze ST and other cardiovascular outcomes if a long-term follow-up was to be considered. Another meta-analysis that compared SES and PES in patients with diabetes with coronary artery diseases showed SES to be associated with a significantly reduced TLR compared to PES.^[41]^ However, the risks of MI, ST, and death were similar. Furthermore, the analysis comparing SES with PES in patients with diabetes again showed SES to be superior compared to PES in terms of TLR and restenosis; however, SES were noninferior to PES in terms of ST, cardiac death, and MI.^[42]^ The meta-analysis published by Yan et al comparing second-generation DES (EES) with first-generation DES showed the former to be highly effective in reducing the risk of MACEs in patients with T2DM.^[43]^ However, their study compared EES with SES separately, which was different when compared to the current study, whereby SES were compared with the other DES combined together (non-SE DES).

In the mixed treatment comparison analysis including 22,844 patients with diabetes obtained from randomized trials, all DES were effective when compared to bare metal stents.^[44]^ Moreover, when SES were compared with PES, they were superior in lowering late lumen loss. However, the current study did not analyze lumen loss. Also, when EES were compared to other DES, EES were associated with better outcomes in these patients with T2DM. In this analysis PES were dominating. Therefore, other DES such as EES that could most probably be more effective than SES could not efficiently show their effectiveness. Lee et al also compared SES with PES in patients with T2DM.^[4]^ Their results showed SES to be superior compared to PES in improving clinical outcomes. However, their study had a follow-up period of only 9 months, whereas the current analysis involved a follow-up period ranging from 6 months to 5 years. Moreover, even if the SORT OUT III substudy showed SES to be associated with better clinical outcomes compared to ZES,^[45]^ only a follow-up period of 18 months was considered.

### 4.1 Novelty

This study is new in several ways. First of all, it is among the first meta-analyses comparing SES with other DES using a large number of patients with diabetes among whom ST is expected to be more prominent after coronary angioplasty. Therefore, this research represents a new idea in clinical medicine. Second, previous meta-analyses comparing different types of DES mainly included patients only from randomized trials. However, this analysis involved a mixture of patients obtained from randomized trials and observational studies representing another new feature. In addition, this meta-analysis compared ST and the other adverse cardiovascular outcomes between 6 months and 5 years follow-up. Total, definite, and probable STs were also analyzed during a follow-up period ranging from 6 months to 2 years, and a long-term follow-up >2 years showing another new feature in this study.

### 4.2 Limitations

This study also has several limitations. First of all, the inclusion of data from observational studies is believed to be associated with a high risk of bias. Therefore, an increased level of heterogeneity was observed when analyzing several subgroups of adverse cardiovascular outcomes. This could also have been due to the comparison of SES with different types of DES (non-SE DES) combined together. Moreover, PES that were dominating among the non-SE DES could also represent a major limitation in this study.

## 5 Conclusions

During this particular follow-up period, SES were not associated with any increase in ST among these patients with T2DM. Mortality and other adverse cardiovascular outcomes were also not significantly different between these 2 groups. Hence, SES should be considered neither superior nor inferior to other DES. They are expected to be equally effective and safe to use in patients with T2DM.
